# Internal mechanism of correlation between angiotensin II gene and serum adiponectin level in patients with cerebrovascular complications of H-type hypertension

**DOI:** 10.5937/jomb0-45532

**Published:** 2024-06-15

**Authors:** Ying Li, Xiufeng Gu, Yun Shi, Jie Li, Shangyu Wen

**Affiliations:** 1 Tianjin Fourth Central Hospital, Department of Cardiology, Tianjin, China

**Keywords:** angiotensin II, H-type hypertension, cerebrovascular complications, serum adiponectin, angiotenzin II, hipertenzija H tipa, cerebrovaskularne komplikacije, serumski adiponektin

## Abstract

**Background:**

The study aimed to explore the correlation between the angiotensin II (Ang II) gene and serum adiponectin expression in patients with cerebrovascular complications of H-type hypertension (HH) and its mechanism.

**Methods:**

A total of 50 cases of outpatient patients in Tianjin Fourth Central Hospital were recruited from January 2022 to June 2023 and rolled into three groups according to their blood pressure and basic information, namely the HH cerebrovascular complications group, the non-H-type hypertension (NHH) group, and the healthy control (HC) group. Peripheral blood samples were taken; one sample was utilized to test for the Ang II gene and the methylation of Ang II, and the other sample was utilized to measure serum adiponectin levels to analyze the relationship between serum adiponectin level and Ang II in patients with cerebrovascular complications of HH.

**Results:**

The ratio of male to female was 8:7 in the group of cerebrovascular complications of HH, and mean systolic blood pressure (SBP) and diastolic blood pressure (DBP) were 167.34 mm Hg and 112.56 mm Hg, respectively. In the NHH group, the mean SBP was 165.89 mm Hg, and the mean DBP was 113.47 mm Hg. The blood pressure of the HC group was in the normal range. The Ang II content was the highest in the group with cerebrovascular complications of HH, followed by the group with NHH, and the lowest in the HC group.

**Conclusions:**

Pyrosequencing chart of patients with cerebrovascular complications of HH showed that the content of deoxyphosphate ribose G was the highest, while the content of A was the highest in NHH patients. Moreover, the serum adiponectin level of patients with HH and NHH was superior to that of the HC group, and the adiponectin level between the former two groups and the HC group differed considerably. Ang II levels were high in patients with cerebrovascular complications of HH and were positively correlated with adiponectin levels. The incidence of cerebrovascular complications of HH may be related to Ang II levels in patients.

## Abstract (sr)

**Uvod:**

Cilj studije je bio da se istraži korelacija između gena angiotenzina II (Ang II) i mehanizam korelacije između gena angiotenzina ii i nivoa serumskog adiponektina kod pacijenata sa cerebrovaskularnim komplikacijama kod hipertenzije tipa H.

**Metode:**

Odabrano je 50 pacijenata sa ambulantnim tretmanom u četvrtoj centralnoj bolnici u Tianjinu od januara 2022. do juna 2023. godine i podeljeno u tri grupe prema krvnom pritisku i osnovnim informacijama, tj. grupu sa cerebrovaskularnim komplikacijama HH tipa, grupu bez H-tipa hipertenzije (NHH) i zdravu kontrolnu grupu (HC). Uzeti su uzorci periferne krvi, pri čemu je jedan uzorak korišćen za testiranje gena angiotenzina II (Ang II) i metilacije Ang II, a drugi uzorak za merenje nivoa serumskog adiponektina, kako bi se analizirao odnos između nivoa serumskog adiponektina i Ang II kod pacijenata sa cerebrovaskularnim komplikacijama HH tipa.

**Rezultati:**

U grupi sa cerebrovaskularnim komplikacijama HH, odnos muškaraca i žena je bio 8:7, a srednji sistolni krvni pritisak (SBP) i dijastolni krvni pritisak (DBP) iznosili su 167,34 mm Hg, odnosno 112,56 mm Hg. U NHH grupi, srednji SBP bio je 165,89 mm Hg, dok je srednji iznosio DBP 113,47 mm Hg. Krvni pritisak u HC grupi bio je u normalnom opsegu. Sadržaj Ang II bio je najviši u grupi sa cerebrovaskularnim komplikacijama HH, zatim u NHH grupi, a najniži u HC grupi.

**Zaključak:**

Grafikon pirosekvenciranja pacijenata sa cerebrovaskularnim komplikacijama HH pokazao je da je sadržaj deoksi-fosfat riboze G bio najviši, dok je sadržaj A bio najviši kod pacijenata sa NHH. Osim toga, nivo serumskog adiponektina kod pacijenata sa HH i NHH je bio viši u poređenju sa HC grupom, a nivo adiponektina kod prve dve grupe i HC grupe se značajno razlikovao. Nivoi Ang II su bili visoki kod pacijenata sa cerebrovaskularnim komplikacijama HH i u pozitivnoj su korelaciji sa nivoima adiponektina. Pojava cerebrovaskularnih komplikacija HH može da bude povezana sa nivoom Ang II kod pacijenata.

## Introduction

H-type hypertension (HH) represents a type of primary hypertension with hyperhomocysteinemia, which is abbreviated as homocysteine (Hcy ≥ 10 μmol/L). Chinese scholars conducted data analysis on hypertensive patients and found that among Chinese adults with hypertension, HH accounted for 75%, among which the proportion of male patients was much superior to that of female patients [1]. The World Health Organization’s diagnostic standard for HH is that fasting plasma cysteine level ≥ 10 μmol/L (the average Hcy level of healthy adults is 5–15 μmol/L), and it belongs to hyper Hcy and hypertension accompanied by high Hcy, which is called »HH« [2]. Studies have shown that the main cause of the increase in Hcy is insufficient vitamin B6, vitamin B12, and folic acid intake. Disruption of methionine circulation in homocysteine anabolism due to insufficient folic acid intake may also be related to genetics. Studies confirmed that the homocysteine level of the MTHFRC677TTT genotype is twice as high as that of the CT/CC genotype, which is due to the long-term eating of methionine-rich protein diet and other factors [3] [4]. Many studies found that Hcy can damage vascular endothelial cells, cause changes in vascular structure, and lead to vascular dysfunction [5]. H2S blocks the Ang converting enzyme (ACE) activity in endothelial cells [6]. Other studies suggested that Hcy induces the synthesis of AT1 receptor-related metalloproteinase-9 and collagen in vascular endothelial cells, leading to vascular remodeling in hypertension [7]. HH is hypertension combined with high homocysteine, which synergically increases the risk of cardiovascular and cerebrovascular events and produces a double effect.

Adiponectin (APN) is a kind of endogenous bioactive peptide or protein secreted by adipocytes. Studies found that serum APN levels in patients with essential hypertension are substantially decreased, which is closely related to blood pressure, obesity, glucose and lipid metabolism, insulin resistance, and inflammatory response. It suggests that hypoAPN may be involved in essential hypertension progression [8]. Related studies found that adiponectin can be utilized as an indicator to judge the condition of hypertension, which is of great significance for controlling essential hypertension [9]. In humans, adiponectin levels have been found to predict the development of cerebrovascular complications. Zhou et al. [10] noted that adiponectin can reduce the adhesion of monocytes, and monocytes can arrange THP-1 cells into rows in the endothelial tissue of human arteries. This adhesion occurs in the early stage of atherosclerotic vascular wall injury. Modern studies pointed out that adiponectin can regulate carbohydrate metabolism. It can also regulate vascular homeostasis via pathways of endothelial cells and regulate subcutaneous inflammatory response, thus intervening in hypertension [11]. Related studies revealed that the APN level of patients with high Hcy is substantially reduced, and after the influence of blood glucose, insulin resistance index, and blood lipid is removed, adiponectin concentration Hcy is negatively correlated [12]. Studies also found that the APN level in patients with HH is substantially reduced than in healthy adults [13].

In human peripheral blood, there were four methylation sites in the Ang-specific receptor promoter region, and the methylation level of the first methylation site in the promoter region was substantially inferior to the healthy group in patients with hypertension. Furthermore, further analysis showed that the hypomethylation state of the methylation site was a predictor of EH. Methylation is a well-studied DNA modification method [14]. At present, researches on the methylation status of susceptibility genes of HH mainly focus on the renin-Ang system, genes related to water and salt metabolism and cell membrane ion transport, adrenergic system and inflammatory factors, and genes related to endothelial function. Some scholars revealed that abnormal DNA methylation changes affect hypertension pathogenesis [15]. Both Ang II and Ang [1] [2] [3] [4] [5] [6] [7] are effectors in the RAS system. The expression and activity of the Ang II receptor in adults are very low. Ang type 1 receptors are commonly expressed in the cardiovascular system and mediate most of the physiological and pathophysiological effects of Ang II. Studies showed that homocysteine (Hcy) can promote Ang II-mediated vascular remodeling, but the mechanism remains unclear [16]. Both 5 μg/mL and 10 μg/mL adiponectin substantially reduced oxidative damage and oxidative stress (ROS) levels after Ang II injury and improved endothelial cell activity

For this reason, patients with HH complicated with cerebrovascular complications were selected as the research object, and the Ang II gene, which has not yet been fully understood, was taken as the breakthrough point to explore the methylation of the promoter region of the Ang II gene. Then, the correlation between the gene and serum adiponectin level in patients with HH complicated with cerebrovascular complications and its mechanism were analyzed to provide a reference for preventing cerebrovascular complications in patients with HH. So, the present study aimed to unravel the internal mechanisms underlying the intricate correlation between the angiotensin II gene and serum adiponectin levels in patients afflicted with cerebrovascular complications arising from H-type hypertension.

## Materials and methods

### Research objects

A total of 56 outpatients in Tianjin Fourth Central Hospital from January 2022 to June 2023 were recruited, including 30 patients with HH and 15 patients with primary non-H-type hypertension (NHH). At the same time, 11 healthy subjects from the physical examination center were randomly selected as controls. All population was classified into HH, NHH, and healthy control (HC) groups. Approved by the corresponding Ethics Committee, this research was implemented.

Inclusion criteria: subjects aged between 20 and 65; the HC group was required to meet the standard of normal blood pressure in *Chinese Guidelines for Prevention and Treatment of Hypertension*, i.e., systolic blood pressure(SBP)<120 mm Hg and diastolic blood pressure(DBP <80 mm Hg; patients in the primary NHH group should meet the requirements of three measurements on different days of SBP>140 mm Hg and/or DBP>90 mm Hg; the subjects had no other acute or chronic diseases; the renal function, blood glucose, and blood lipid of the subjects were within the normal range; the study subjects volunteered to participate, cooperated with various examinations, and signed the informed consent.

Exclusion criteria: the patient was suspected or had been diagnosed with secondary hypertension; patients took VitB12, folic acid, and other drugs affecting the metabolism within one month; patients suffering from other diseases, such as infectious diseases, other cardiovascular diseases, and endocrine diseases; patients participated in other researchers at the same time; patients with a history of drinking or smoking; patients did not cooperate with experimental research.

### Serum sample extraction and detection

Peripheral venous blood was extracted from the subjects (fasting state) in the morning, 3 mL of which was kept in the anticoagulant tube with matching reagents. The blood sample was fully mixed with anticoagulant and stored in a refrigerator at -80°C for DNA extraction and gene analysis. The other was restored at -20°C for serum adiponectin levels.

### Methylation level in Ang II gene promoter region

First, the location of the CpG island in the Ang II gene promoter region, which was located -380 to -1,500 bp upstream of the transcription start point, was analyzed. Then, the sequence to be extracted was determined, and the blood genome extraction kit was utilized for DNA extraction. Finally, according to the determined sequence, polymerase chain reaction (PCR) primers were designed employing certain software. PCR amplification required a PCR reaction system, which included template DNA, primers, dNTO, Taq Buffer, and Taq enzyme. The amplification reaction needed to go through four steps of pre-denaturation (95°C, 3 min), denaturation (94°C, 30 sec), annealing (57°C, 30 sec), extension (72°C, 45 sec), and cycle 35 times. The methylation level of the amplified product fragments was detected, and the pyrosequencing method was utilized to analyze the methylation level of the Ang II gene promoter region.

### Detection of serum adiponectin

First, the blood sample that was placed at -20°C was taken out and melted at room temperature, and the kit was prepared in advance. Secondly, the standard hole (added with dilution), test sample hole (added with test sample), and blank control hole were set up to obtain the standard curve. Then, the kit was sealed and incubated at 37°C. After the warm bath, the sealing plate film was removed, and the reaction plate was washed thoroughly. HRP-conjugated reagents (labeled anti-human antibodies) were added to each well. The chromo graphic agent was added in each hole and shaken to make it fully mixed, avoiding light to make it chromo graphic. Finally, the terminating solution was applied, and then absorbance was measured in sequence by wavelength, with a blank hole as zero control.

### Results calculation and judgment

All values were calculated by subtracting the blank values. The standard curve was drawn on semi-logarithmic paper with OD values of 4,000, 2,000, 1,000, 500, 250, 125, 62.5, and 0 ng/L. The line shall be a smooth curve with the concentration as the axis (the logarithm axis) and the value as the axis (the linear axis). The corresponding serum adiponectin content was detected on this curve according to the sample value.

### Correlation between changes of Ang II methylation and serum adiponectin

According to the degree of methylation of Ang II, the subjects were rolled into three groups, which were the persistent hypomethylation state group (n=28), the induced demethylation state group (n=13), and the hypermethylation state group (n=9). The adiponectin level of each group was determined.

### Statistical analysis

Employing SPSS 21.0, the methylation level of the target gene was expressed as a percentage, and each variable of measurement data was recorded as mean plus or minus standard deviation. The normal distribution and variance homogeneity tests were carried out on the data. One-way analysis of variance was adopted for comparison among groups, and the least considerable difference method was utilized for pair comparison between groups. *P*<0.05 indicated a statistical difference.

## Results

### Comparative analysis of general data

In the HH group, there were 30 patients, of whom the male-to-female ratio was 8:7. The average SBP of the patients was 167.34 mm Hg, and the DBP was 112.56 mm Hg. There were 15 patients in the NHH group, of which the male-to-female ratio was 13:12. The average SBP of this group was 165.89 mm Hg, and the average DBP was 113.47 mm Hg. The HC group was abbreviated as the healthy group. There were 11 healthy persons with a male-to-female ratio of 6:5. Their average SBP was 111.34 mm Hg, and their average DBP was 74.87 mm Hg (Table 1).

**Table 1:** General information of research subjects.

	HH<br>cerebrovascular<br>complications<br>group (n=30)	NHH<br>group<br>(n=15)	HC group<br>(n=11)
Gender<br>(Male/Female)	16/14	13/12	6/5
Average age<br>(years)	56.21	57.32	56.34
BMI (kg/m^2^)	24.87	24.19	22.89
Mean SBP<br>(mm Hg)	167.34	165.89	111.34
Mean DBP<br>(mm Hg)	112.56	113.47	74.87

### Electrophoresis observation

**Figure 1:**
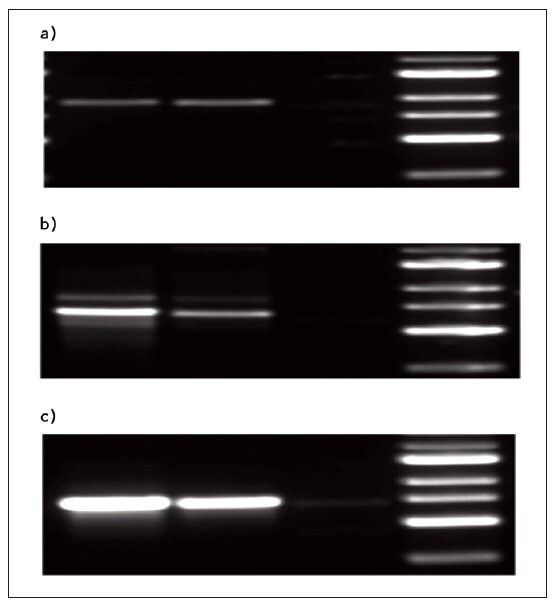
**(a)** Electrophoresis diagram of healthy patients. **(b)** Electrophoresis diagram of patients in NHH group. **(c)** Electrophoresis of patients with HH cerebrovascular complicationsgroup.

The serum Ang II in the three groups was observed by electrophoresis. The highest Ang II content was found in the group with HH and cerebrovascular complications, followed by the group with NHH, and the lowest Ang II content in the HC group (Figure 1a–Figure 1c).

### Ang II gene promoter region methylation level detection results

#### I. Pyrosequencing diagram of Ang II gene promoter region

**Figure 2:**
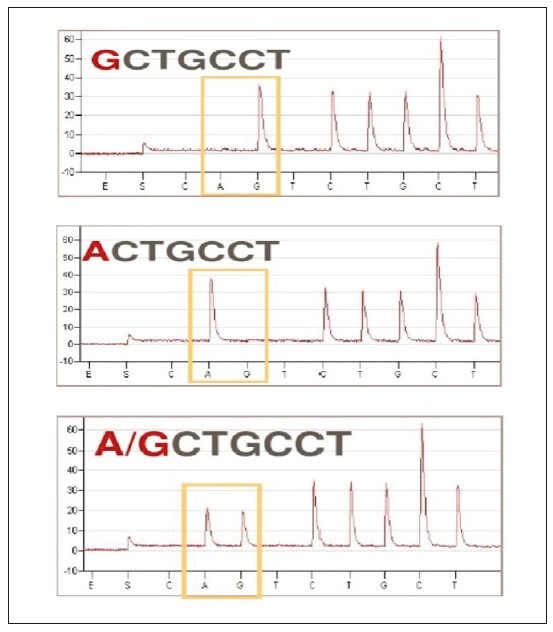
**(a)** Sequencing diagram of methylation level in patients with HH and cerebrovascular complications. **(b)** Sequencing diagram of methylation level in patients with NHH. **(c)** Sequencing diagram of the methylation level of healthy control patients.

Pyrosequencing of patients in the H-type hypertensive cerebrovascular complications group showed that the content of deoxyphospho ribose G was the highest, and the content of deoxyphospho ribose A was the highest in patients with NHH. For healthy examinees, the proportions of deoxyphospho ribose A and G in the pyrosequencing chart were the same (Figure 2 (a, b, c)).

#### II. Statistics of methylation level in Ang II gene promoter region

**Figure 3:**
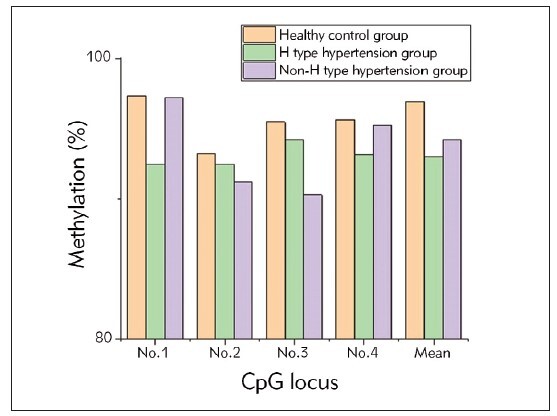
The methylation level of CpG site in Ang II gene promoter region of three groups of patients.

There were four CpG methylation islands in the Ang II gene promoter region in the HC group, HH cerebrovascular complications group, and NHH group, and the difference in methylation of these four sites was not statistically considerable (*P*>0.05) (Figure 3).

### Comparison of three groups of serum adiponectin indexes

**Figure 4:**
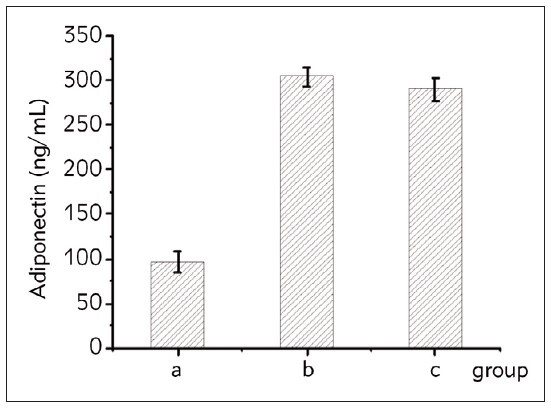
Comparison of serum adiponectin levels in the three groups of subjects. Note: a represented the HC group, b represented the H-type high blood cerebrovascular complications group, and c represented the NHH group.

Figure 4 showed that the levels of adiponectin in the serum of patients with the HH and NHH groups were superior to those of the HC group. The comparison of adiponectin levels between the first two groups and the HC group was statistically considerable (*P*<0.05).

### Correlation between Ang II gene methylation changes and serum adiponectin levels

**Figure 5:**
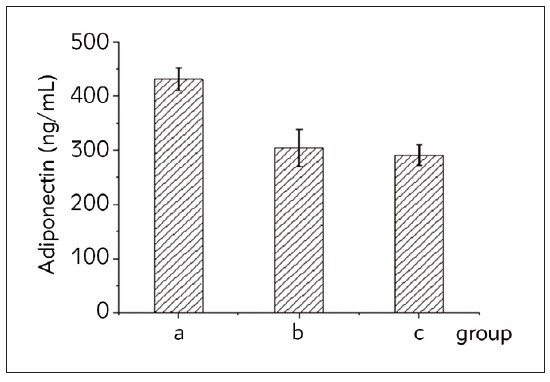
The relationship between Ang II gene methylation level and adiponectin. Note: a was the continuous hypomethylation state group (n=28), b was the induced demethylation state group (n=13), and c was the hypermethylation state group (n=9).

According to the different methylation levels of the Ang II gene, the patients were assigned into three groups. The adiponectin levels in the serum of these three groups were negatively correlated with the degree of methylation (Figure 5).

## Discussion

The present study aimed to unravel the internal mechanisms underlying the intricate correlation between the angiotensin II gene and serum adiponectin levels in patients afflicted with cerebrovascular complications arising from H-type hypertension.

In this study, three groups were analyzed based on hypertension subtypes: H-type hypertension with cerebrovascular complications (HH), non-H-type hypertension with cerebrovascular complications (NHH), and a healthy control group (HC). The demographic characteristics revealed variations in group sizes and gender distributions. HH had 30 patients (male:female ratio 8:7), NHH had 15 patients (male:female ratio 13:12), and HC had 11 individuals (male:female ratio 6:5). In the study of clinical patient data, it was found that among Chinese adult hypertension patients, HH accounted for more than half of all hypertension patients, which was about 75%, and the proportion of male patients was much superior to that of female patients [17].

The blood pressure profiles reflected the severity of hypertension in the studied groups. HH exhibited the highest average systolic blood pressure (SBP) at 167.34 mm Hg and diastolic blood pressure (DBP) at 112.56 mm Hg, followed by NHH with an average SBP of 165.89 mm Hg and DBP of 113.47 mm Hg. The HC group, representing healthy individuals, had considerably lower average SBP (111.34 mm Hg) and DBP (74.87 mm Hg). Niu et al. [18] study results showed significant changes in blood pressure and heart rate of patients with hypertensive cerebrovascular disease.

Serum Ang II content, assessed through electrophoresis, revealed a distinct pattern. The HH group with cerebrovascular complications exhibited the highest Ang II content, followed by NHH, and the lowest levels were observed in the HC group. This suggests a potential association between elevated Ang II levels and the presence of cerebrovascular complications in hypertensive patients. These findings are consistent with previous studies [19] [20] [21] [22].

Pyrosequencing of the Ang II gene promoter region indicated variations in deoxyphospho ribose content. In HH patients, deoxyphospho ribose G was highest, while in NHH patients, deoxyphospho ribose A was predominant. Healthy individuals displayed an equal proportion of deoxyphospho ribose A and G. However, the statistical analysis of methylation levels at four CpG islands in the Ang II gene promoter region did not reveal significant differences among the HC, HH, and NHH groups (P>0.05).

Adipocytes release a cytokine called adiponectin, which has anti-inflammatory, anti-atherosclerotic, and endothelium-protective properties [23]. Serum adiponectin levels demonstrated a significant difference among the groups. HH and NHH groups showed higher levels than the HC group, with statistical significance (P<0.05) in the comparison between the hypertensive and healthy control groups. This indicates a potential association between hypertension and increased adiponectin levels. For the first time, Ruan et al. [24] showed in their study that adiponectin and visfatin are risk factors for stroke in patients with high blood pressure. Inconsistent with our study, Choi et al.’s [25] study showed that cerebral infarction patients had significantly lower serum adiponectin levels than control patients. According to this, a reduction in adiponectin levels is one of the main contributing factors to the onset and course of vascular illnesses, such as acute cerebral infarction [25]. Also, In Niu et al. [18] study, adiponectin in patients with hypertensive cerebrovascular disease was significantly reduced, while the hs-CRP and sICAM-1 content was significantly increased.

The inconsistency of the results obtained from our study regarding the increase of adiponectin can be attributed to the influence of adiponectin on other factors. For example, according to Lindstrom et al. [19], individuals who have had their condition for more than ten years have greater blood levels of ADPN, which is related to the decline in renal function as the disease progresses. Studies have shown a correlation between blood glucose management, age, cholesterol, and the length of diabetes. A marker for coronary heart disease, hs-CRP, has been shown to negatively correlate with the amount of ADPN in subcutaneous adipose tissues in heart disease patients. Some studies indicate that people with cerebrovascular illness have greater levels of sICAM-1 [20].

The present study further explored the correlation between Ang II gene methylation and serum adiponectin levels. Three groups were categorized based on different methylation levels, revealing a negative correlation between the degree of Ang II gene methylation and adiponectin levels. This suggests that higher methylation levels may be associated with lower adiponectin levels in the serum. In line with our study, many studies found that Ang II is involved in a series of physiological processes, namely regulation of the renin-Ang system, water and salt metabolism, cell membrane example transport, as well as inflammatory factors and endothelial cell function [21] [22]. A study by a researcher in the United States found that there were four methylation sites in the Ang-specific receptor promoter region in human peripheral blood. The methylation level at the first methylation site of the promoter region was substantially lower in hypertensive patients than in healthy control patients [23].

Numerous research studies have examined the complex interaction between serum adiponectin levels, methylation of the Ang II gene, and cerebrovascular problems in patients with H-type hypertension [24] [25] [26] [27]. Although the search results are insightful into Ang II signaling and its relationships, there isn’t a clear discussion of a direct connection between Ang II gene methylation, serum adiponectin, and H-type hypertension-specific cerebrovascular problems.

In conclusion, we found that patients with cerebrovascular complications of H-type hypertension (HH) and non-H-type hypertension (NHH) had different patterns of deoxyphosphate ribose G and A content in the angiotensin II gene promoter region, as indicated by the pyrosequencing chart [28] [29] [30] [31]. Significant variations were seen between the HH and NHH groups’ blood adiponectin levels, which were notably higher in these groups than in the healthy control (HC) group. Additionally, our results showed a favorable association between adiponectin and angiotensin II (Ang II) levels, which was especially significant in individuals with HH-related cerebrovascular problems. This implies that there may be a connection between high Ang II levels and the prevalence of vascular problems in patients with H-type hypertension [32] [33]. To sum up, our research illuminates the underlying processes that connect the pathophysiology of cerebrovascular problems in H-type hypertension, serum adiponectin levels, and the angiotensin II gene. These findings will be useful for future investigation and clinical considerations.

## Dodatak

### Conflict of interest statement

All the authors declare that they have no conflict of interest in this work.
